# Supplementary material for: The Glycolytic Metabolite Methylglyoxal Covalently Inactivates the NLRP3 Inflammasome
Source: bioRxiv. 2024 Apr 20:2024.04.19.589802. Preprint. [Version 1] doi: 10.1101/2024.04.19.589802 (PMC11042358; doi:10.1101/2024.04.19.589802)
Supplement: 1 [file NIHPP2024.04.19.589802v1-supplement-1.pdf]

bioRxiv preprint doi: https://doi.org/10.1101/2024.04.19.589802; this version posted April 20, 2024. The copyright holder for this preprint (which was not certified by peer review) is the author/funder, who has granted bioRxiv a license to display the preprint in perpetuity. It is made available under aCC-BY 4.0 International license.

Table S1: Compounds

**Compound**	**Source**	**Catalog Number**
ML385	Cayman Chemical	21114-5

Table S2: Antibodies for Western Blotting

Protein	Supplier	Catalog Number	Host Species	Dilution
NLRP3 (NBD)	Cell Signaling	15101S	Rabbit	1:1000 (BSA)
NLRP3 (PYN)	Adipogen	AG-20B-0014-C100	Mouse	1:1000 (BSA)
Tubulin	Sigma	T6557	Mouse	1:2000 (BSA)
HIS-Tag	Santa Cruz	Sc-8036	Mouse	1:1000 (BSA)
FLAG	Sigma	F1804	Mouse	1:1000 (Milk)
IL-1β	GeneTex	GTX74034	Rabbit	1:1000 (BSA)
GFP	Abcam	Ab290	Rabbit	1:1000 (BSA)
ASC	Cell Signaling	13833S	Rabbit	1:1000 (BSA)
CASP1	Cell Signaling	3866S	Rabbit	1:1000 (BSA)
NEK7	Cell Signaling	3057S	Rabbit	1:1000 (BSA)

Table S3: qPCR Primers

**Gene**	**Forward**	**Reverse**
PGK1	CAAGCTGGACGTTAAAGGGA	CAAGCTGGACGTTAAAGGGA
NLRP3	GATCTTCGTTGCGATCAACA	GGGATTCGAAACACGTGCATTA
Pro-IL-1B	AGCTCGCCAGTGAAATGATG	GGTGGTCGGAGATTCGTAGC
NQO1	GCCTCCTTCATGGCATAGTT	GGACTGCACCAGAGCCAT
HMOX1	GAGTGTAAGGACCCATCGGA	GCCAGCAACAAAGTGCAAG
GAPDH	AATGAAGGGGTCATTGATGG	AAGGTGAAGGTCGGAGTCAA
RiboPro	CGTCGCCTCCTACCTGCT	CCATTCAGCTCACTGATAACCTTG

bioRxiv preprint doi: https://doi.org/10.1101/2024.04.19.589802; this version posted April 20, 2024. The copyright holder for this preprint (which was not certified by peer review) is the author/funder, who has granted bioRxiv a license to display the preprint in perpetuity. It is made available under aCC-BY 4.0 International license.

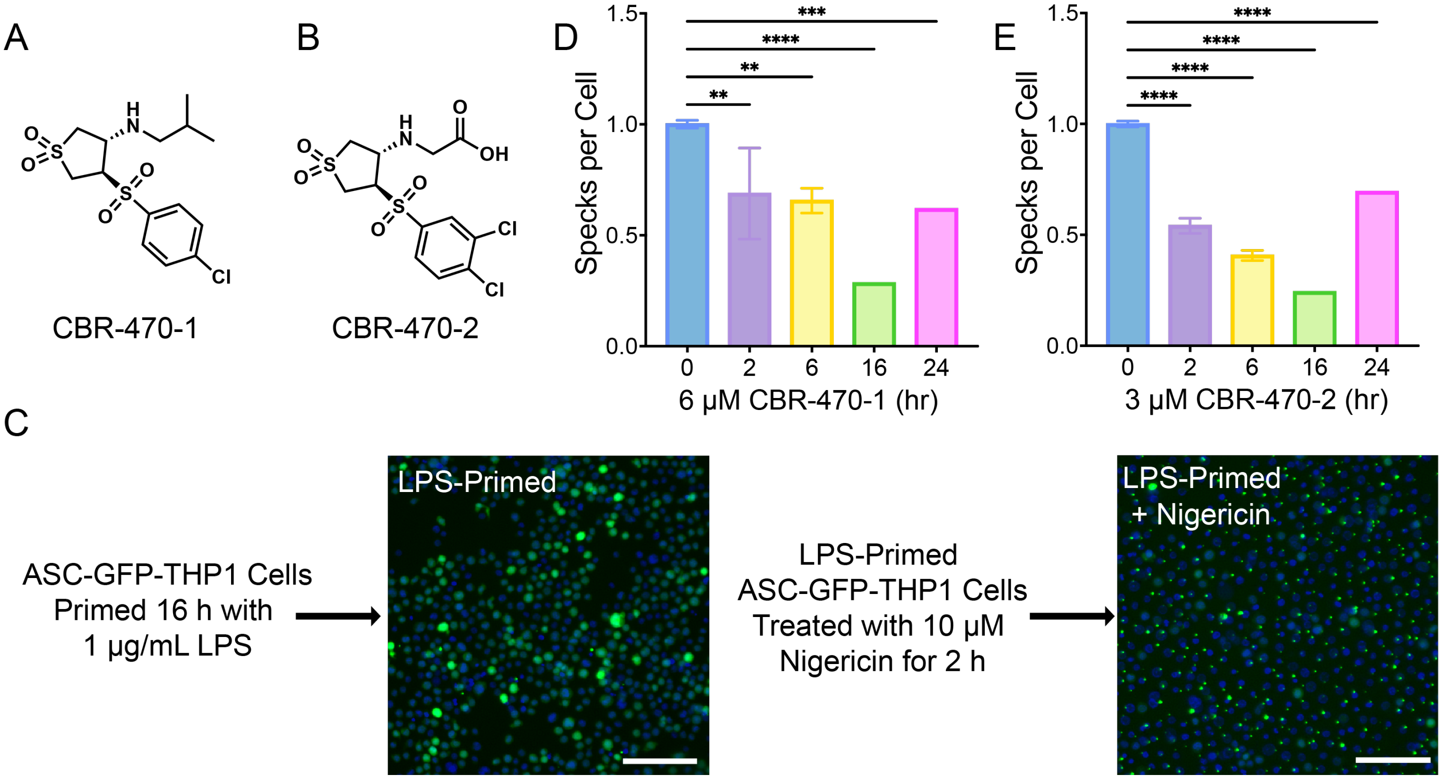

Supplemental Figure 1. CBR-470-1 and CBR-470-2 display time dependent inhibition at lower concentrations.
Structures of CBR-470-1 (A) and CBR-470-2 (B). (C) Schematic with representative images of ASC-GFP Speck formation induced by LPS and Nigericin. Scale bar = 100 μM. (D,E) Number of ASC-GFP specks per cell in THP1-ASC-GFP cells pretreated with CBR-470-1 at 6 μM (D) and CBR-470-2 at 3 μM (E) for 0-24 h. Error bars show SEM for n = 3 replicates.

bioRxiv preprint doi: https://doi.org/10.1101/2024.04.19.589802; this version posted April 20, 2024. The copyright holder for this preprint (which was not certified by peer review) is the author/funder, who has granted bioRxiv a license to display the preprint in perpetuity. It is made available under aCC-BY 4.0 International license.

A

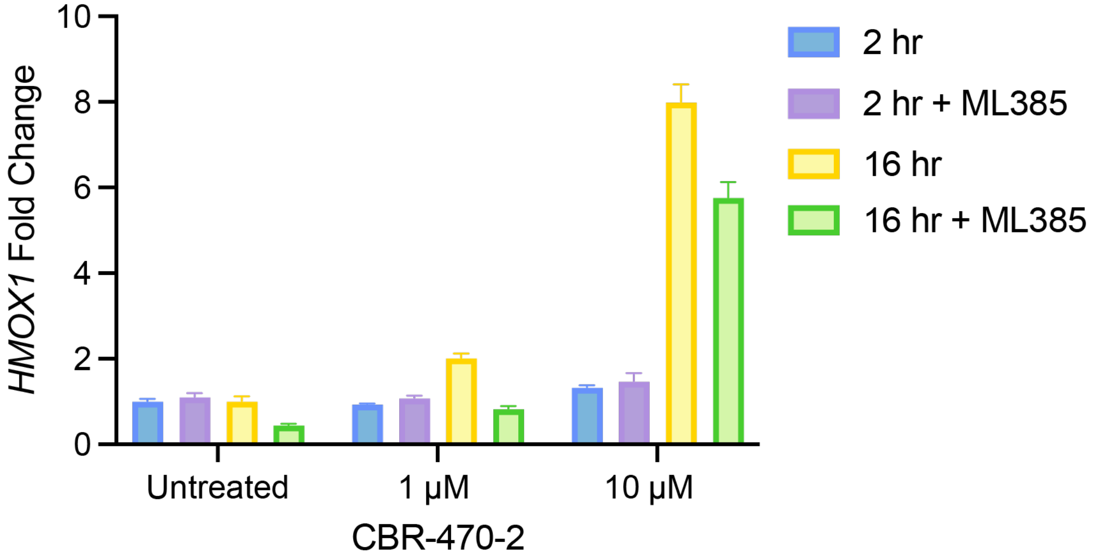

Supplemental Figure 2. CBR-470-2 induces NRF2 target gene expression at 16 h but not 2 h. (A) Relative transcript level for *HMOX1* as measured by qPCR from WT THP1 cells pre-treated with or without 10 μM ML385 for 30min and then treated CBR-470-2 for 2 or 16 h. Error bars show SEM for n = 3 replicates.

bioRxiv preprint doi: https://doi.org/10.1101/2024.04.19.589802; this version posted April 20, 2024. The copyright holder for this preprint (which was not certified by peer review) is the author/funder, who has granted bioRxiv a license to display the preprint in perpetuity. It is made available under aCC-BY 4.0 International license.

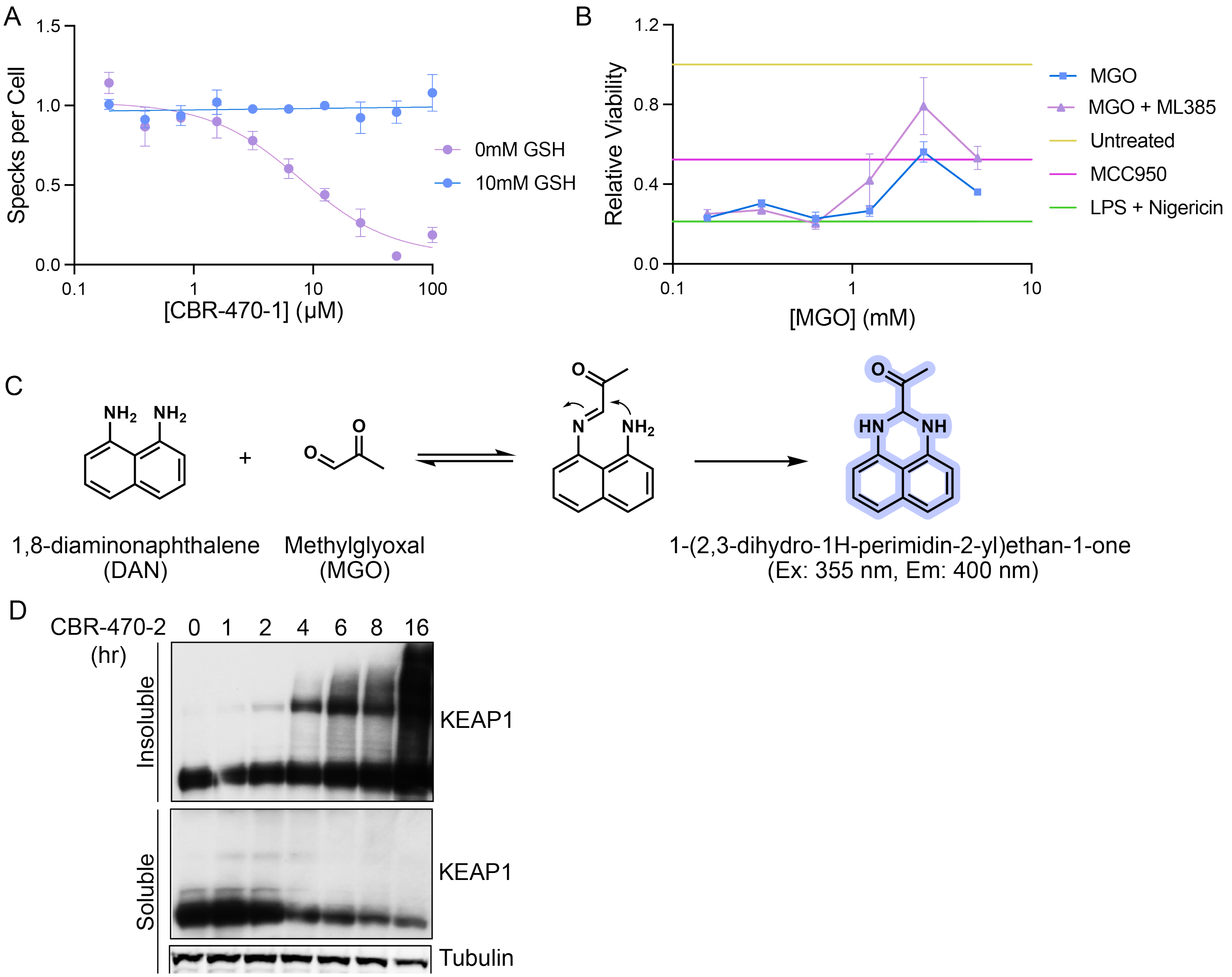

Supplemental Figure 3. Inhibition or knockdown of PGK1 induces methylglyoxal accumulation.
(A) Number of ASC-GFP specks per cell in THP1-ASC-GFP cells pre-treated with 0 or 10 mM GSH and then treated in dose response with CBR-470-1. Error bars show SEM for n = 3 replicates. (B) Relative viability of LPS-primed (1 μg/mL, 16 h) WT THP1 following NLRP3-mediated pyroptotic cell death induced by Nigericin (10 μM, 2.5 h), pretreated with or without 10 μM ML385 for 30 min and then with MGO (2 h) in dose response, or with 10 μM MCC950 (2 h). Error bars show SEM for n = 3 replicates. (C) 1,8-Diaminonapthalene reaction with MGO to form fluorescent compound. (D) Western blot of soluble and insoluble KEAP1 and Tubulin from WT THP1 cells treated with 50μM CBR-470-2 for 1 to 16 h.

bioRxiv preprint doi: https://doi.org/10.1101/2024.04.19.589802; this version posted April 20, 2024. The copyright holder for this preprint (which was not certified by peer review) is the author/funder, who has granted bioRxiv a license to display the preprint in perpetuity. It is made available under aCC-BY 4.0 International license.

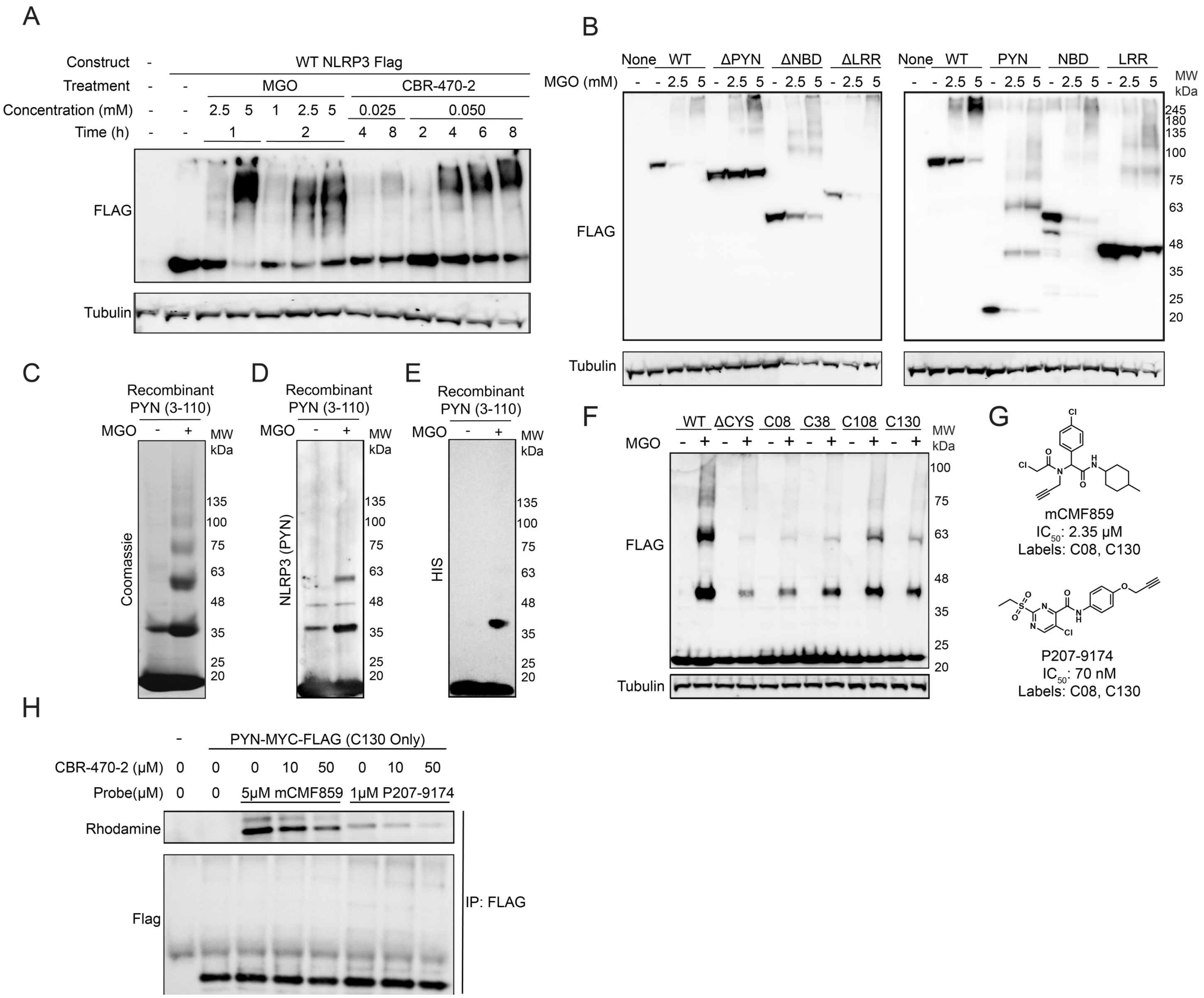

Supplemental Figure 4. CBR-470-2 and MGO induce covalent crosslinks of NLRP3 Pyrin domain cysteines.

(A) Western blot for FLAG and Tubulin in HEK293T cells overexpressing the indicated FLAG-Tagged NLRP3 treated with CBR-470-2 or MGO at the indicated concentrations and timepoints. (B) Western blot for FLAG and Tubulin in HEK293T cells overexpressing the indicated FLAG-Tagged NLRP3 domain constructs treated with 0, 2.5 or 5 mM MGO for 1 h. (C-E) Coomassie Blue stain (C) or Western blots of NLRP3 (PYN) (D) and HIS-TAG (E) from recombinant NLRP3 PYN (3-110, C08S, C38S) treated with 5 mM MGO at 4 °C for 1 h. (F) Western blot of FLAG from HEK293T cells overexpressing NLRP3-FLAG PYN domain constructs with cysteines mutated and individually reintroduced, treated with or without 5 mM MGO for 1 h. (G) Structures and activities of mCMF859 and P207-9174. (H) Anti-FLAG Western blot and rhodamine imaging of FLAG immunoprecipitated material after in situ treatment of HEK293T cells expressing FLAG-tagged PYN domain C130 only construct treated with CBR-470-2 (6h) and then 1 μM P207-9174 or 5 μM mCMF859 (1 h).

bioRxiv preprint doi: https://doi.org/10.1101/2024.04.19.589802; this version posted April 20, 2024. The copyright holder for this preprint (which was not certified by peer review) is the author/funder, who has granted bioRxiv a license to display the preprint in perpetuity. It is made available under aCC-BY 4.0 International license.

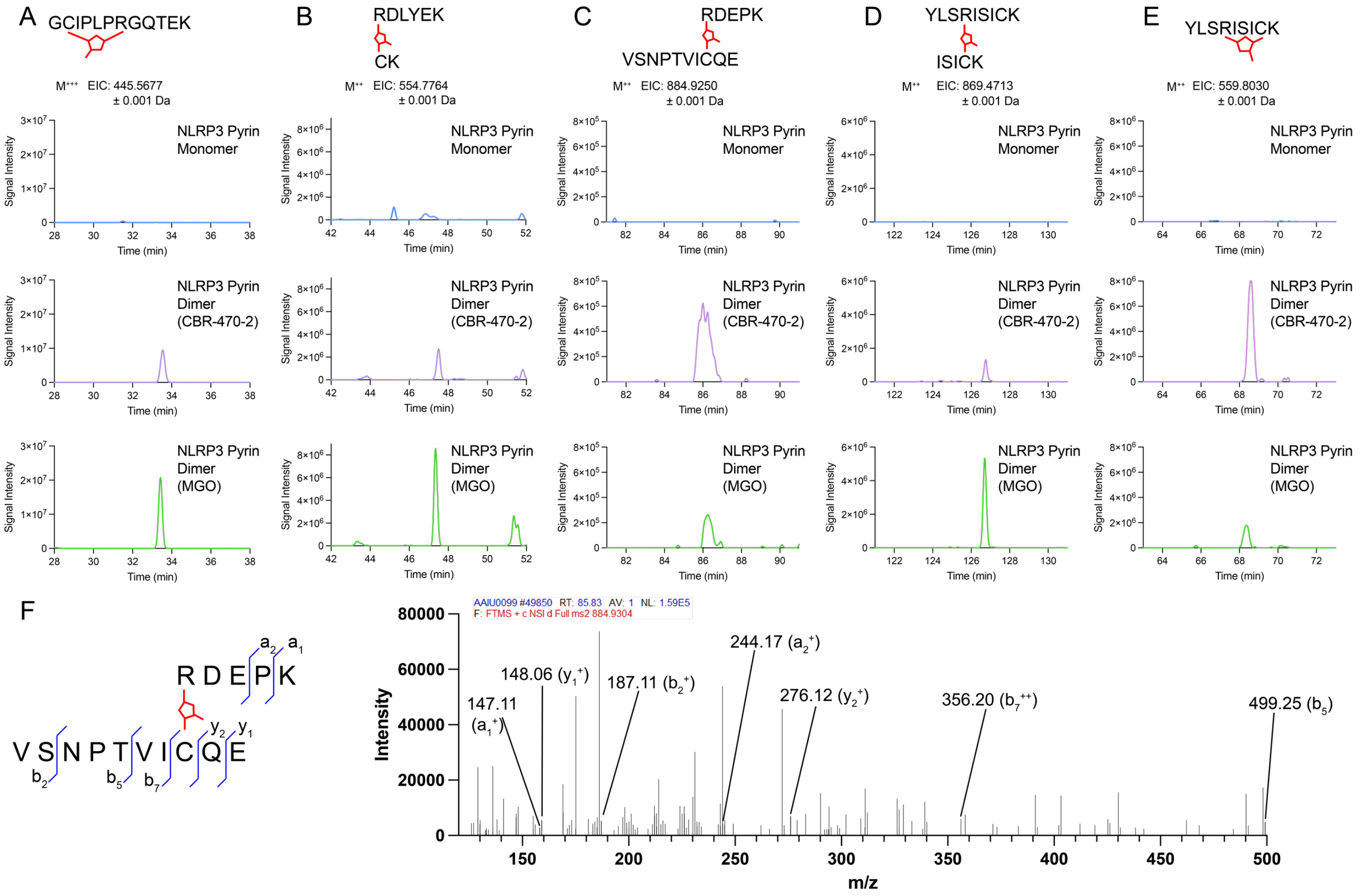

Supplemental Figure 5. CBR-470-2 and MGO induce MICA crosslinks among NLRP3 Pyrin domains. (A-E) EICs from LC–MS/MS analyses of gel-isolated and digested HMW-PYN (CBR-470-2 and MGO-induced) and monomeric PYN for intramolecular C38-R43 (A), C08-R81 (B), C108-R89 (C), intermolecular C130-R126 (D), and intramolecular C130-R126 (E) crosslinked peptides. (F) Annotated MS2 spectrum from the crosslinked C108-R89 PYN peptide.
